# The genetically encoded biosensor HyPer7 enables in-line monitoring of H_2_O_2_ accumulation dynamics in the methylotrophic yeast *Komagataella phaffii*

**DOI:** 10.1093/femsyr/foaf070

**Published:** 2025-11-22

**Authors:** Victor Mendes Honorato, Jennifer Staudacher, Mikael Molin, Brigitte Gasser

**Affiliations:** Department of Biotechnology and Food Science, Institute of Microbiology and Microbial Biotechnology, BOKU University, 1190 Vienna, Austria; Department of Biotechnology and Food Science, Institute of Microbiology and Microbial Biotechnology, BOKU University, 1190 Vienna, Austria; Austrian Centre of Industrial Biotechnology (ACIB), 1190 Vienna, Austria; Division of Systems and Synthetic Biology, Department of Biology and Biological Engineering, Chalmers University of Technology, 405 30 Gothenburg, Sweden; Department of Biotechnology and Food Science, Institute of Microbiology and Microbial Biotechnology, BOKU University, 1190 Vienna, Austria; Austrian Centre of Industrial Biotechnology (ACIB), 1190 Vienna, Austria

**Keywords:** HyPer7, H_2_O_2_ biosensor, methanol metabolism, methylotrophic yeast, oxidative stress, alcohol oxidase

## Abstract

In methylotrophic yeasts such as *Komagataella phaffii* (syn *Pichia pastoris*), the initial step of methanol metabolism by alcohol oxidase (Aox) generates hydrogen peroxide (H_2_O_2_) as a potentially toxic byproduct. Introduction of the ratiometric, genetically encoded fluorescent H_2_O_2_ biosensor HyPer7 in combination with cultivation in a microbioreactor allowed for the first time to *in vivo* determine H_2_O_2_ dynamics upon methanol utilization (MUT). *In line* monitoring of H_2_O_2_ during growth on glucose or methanol revealed a general increase in biosensor oxidation on methanol, with significant oxidation peaks shortly after methanol addition. HyPer7 also detected low endogenous H_2_O_2_ levels occurring during respiratory growth in *K. phaffii* and its signal responded to both external oxidants and reductants. In strains with different MUT phenotypes (*K. phaffii* deleted for *aox1* and/or *aox2*), HyPer7 demonstrated that H_2_O_2_ production is mainly due to Aox1 activity, and explained why strains possessing only Aox2 (Mut^S^) have superior growth and production capacities compared to the wild-type. In conclusion, we present the first application of an H_2_O_2_ biosensor in *K. phaffii*, offering new insights into methanol metabolism and oxidative stress. The findings hold promise for optimizing yeast cell factories and developing more sustainable production processes with reduced oxidative stress in the future.

## Teaser

Introducing the genetically encoded H_2_O_2_ biosensor HyPer7 in the yeast *Komagataella phaffii* reveals real-time oxidative stress dynamics during methanol metabolism, and improves understanding for enhanced yeast-based production processes.

## Introduction

Redox biology necessitates reliable methods to measure *in vivo* fluctuations of reactive oxygen species (ROS). The term ROS depicts various chemically reactive derivatives of molecular oxygen, such as superoxide anion, hydrogen peroxide (H_2_O_2_), hydroxyl radical, and singlet oxygen, which originate from diverse cellular sources, including the mitochondria, peroxisomes, and endoplasmic reticulum (ER; Holmström and Finkel 2014, Sies and Jones 2020). In the cell, ROS act as important signaling molecules for diverse cellular functions, however, they are also causing oxidative damage to biomolecules including DNA, proteins, and lipids, leading to cellular dysfunction and are associated with various human diseases such as cancer, neurodegenerative disorders, and aging (Di Marzo et al. 2018, Lennicke and Cochemé 2021). To prevent oxidative stress and damage, cells have developed sophisticated antioxidant defense systems to regulate ROS levels.

Studying specific ROS such as H_2_O_2_ within living organisms still presents a variety of challenges (Murphy et al. 2022). Fluorescent dyes such as dihydroethidium, dichlorofluorescein, or dihydrorhodamine, which become fluorescent upon reaction with ROS, have commonly been used to detect ROS species. However, these redox sensitive dyes possess limitations, particularly related to their lack of both specificity and permeability (Kalyanaraman et al. 2012, Winterbourn 2014). In the last decades, genetically encoded fluorescent biosensors have been developed that can specifically detect H_2_O_2_ in living cells with temporal and ideally also spatial resolution i.e. within distinct cellular compartments (Kostyuk et al. 2020). These sensors typically consist of a fluorophore linked to a ROS-reactive moiety, allowing for real-time visualization and measurement of ROS levels in living biological systems. Upon reaction with ROS, the fluorophore undergoes a change in fluorescence intensity or wavelength, providing a readout that correlates with ROS concentration (Smolyarova et al. 2022).

For measuring H_2_O_2_, there are two main exponents, the cpYFP-based HyPer family of biosensors and the roGFP2-based biosensors. Very recently, also HaloTag combined with Janelia Fluor rhodamine dye (oROS-HT635) was described for far-red H_2_O_2_ imaging (Lee et al. 2025). The HyPer family is characterized by a fusion between circularly permutated yellow fluorescent protein (cpYFP) and a H_2_O_2_-sensitive bacterial transcription factor OxyR (Belousov et al. 2006). Its mechanism relies in having ratiometric readouts due to a dual fluorescent profile whenever the biosensor is reduced or oxidized. Briefly, upon oxidation of OxyR, the formation of an intramolecular disulfide bond culminates in a conformational change in the cpYFP, increasing the excitability at 488 nm and decreasing it at 405 nm. When the fusion protein is reduced, the intensity of fluorescence is higher when excited at 405 nm than at 488 nm. This allows the measurement of the probe’s redox state by calculating the ratio between the 488 nm and 405 nm fluorescence signals (488 nm/405 nm). HyPer7, which is the latest member of the HyPer family, has overcome the pH-sensitivity associated with previous HyPer-sensors (Pak et al. 2020) and consists of a circularly permuted green fluorescent protein (GFP) integrated into the ultrasensitive OxyR domain from *Neisseria meningitidis*. The other series are the roGFP2-based biosensors, which work in a very similar way, with the main difference being that sensor oxidation initiates by the oxidation of a peroxiredoxin (such as Prx1 or Tsa2) that transfers the oxidation to the fused roGFP2 via thiol exchange (Morgan et al. 2016). Both types of sensors have been successfully applied in mammalian cells, plants and the model yeast species *Schizosaccharomyces pombe* and *Saccharomyces cerevisiae* (De Cubas et al. 2021, Gast et al. 2021, Kritsiligkou et al. 2021, Gast et al. 2022). However, so far they have not been tested for industrially relevant, nonconventional yeast species. Monitoring H_2_O_2_ is of particular relevance in methylotrophic yeasts such as *Komagataella phaffii* (syn *Pichia pastoris*), as this toxic byproduct is generated by alcohol oxidase (Aox) in the initial step of methanol utilization (MUT; Cregg et al. 1989). Accumulation of ROS was associated with low viability of *K. phaffii* in methanol-based bioprocesses (Xiao et al. 2006). Furthermore, *K. phaffii* strains with increased capacity for production of secreted recombinant proteins showed increased ROS accumulation (Delic et al. 2012), as H_2_O_2_ is also generated during the oxidative folding of proteins in the ER. However, so far ROS was only measured unspecifically with small redox-active dyes.

To close this gap, we expressed and evaluated genetically encoded H_2_O_2_ biosensors in *K. phaffii*, shedding light on the dynamics of H_2_O_2_ generation and decomposition in different strains and physiological contexts.

## Materials and methods

### Plasmid construction

All plasmid construction was performed in DH10B *Escherichia coli*. HyPer7 reference sequence (Pak et al. 2020) was codon optimized for yeast expression, synthesized, and ordered from Twist Biosciences. roGFP2-Tsa2dCR plasmid was ordered (Plasmid #83238, Addgene), the sequence of interest was amplified and cloned in appropriate in-house vector. roGFP2-Prx1 was generated by cloning the roGFP2 sequence to the Prx1 (from *S. cerevisiae*) gene via Golden Gate Assembly. Plasmid generation was performed with the GoldenPiCS system, a modular GoldenGate based cloning platform (Prielhofer et al. 2017). Therefore, silent mutations were introduced to avoid recognition by the BsaI and BpiI restriction enzymes used in this system.

The biosensors were then cloned in the final expression vector BB3eH, which harbors the HygMX resistance marker and the *K. phaffii ENO1* region for genome integration in. For constitutive and strong expression of biosensors genes, it was designed to be under control of the TEF promoter (Prielhofer et al. 2017). The final plasmid was confirmed by DNA sequencing.

Aox genes knockout were performed following a *K. phaffii* CRISPR/Cas9 editing strategy (Gassler et al. 2019). CRIS*Pi* plasmid targeting the *AOX1* native locus was previously described (Gassler et al. 2020). Regions flanking the *AOX1* gene were amplified from wild type CBS7435 via PCR, serving as homologous recombination donor fragments during subsequent transformation. The same strategy was performed for knockout of *AOX2*.

### Strain construction

*Komagataella phaffii* strain CBS7435 was used as the main strain for our experiments. Both HyPer7 expression and gene knockouts were integrated into the *K. phaffii* genome using electrocompetent CBS7435 as previously described (Gasser et al. 2013). Preceding transformation, 1 µg of BB3eH-HyPer7 was linearized with *SmaI* to allow for genome integration into the *ENO1* locus (New England Biolabs). Transformants were selected in YPD plates containing 200 µg/ml of hygromycin, after 48 h of incubation at 30°C single clones were picked and confirmed via colony PCR. The resulting strain, CBS7435-HyPer7, was subsequently used in the creation of knockout strains.

For CRISPR/Cas9-directed knockouts, 5 µg of linearized donor fragments and 1 µg of CRIS*Pi* plasmid were concomitantly used in for transformation. First, CBS7435-HyPer7∆*aox1* (MutS) were generated and confirmed using gene specific and flanking regions PCR. This was followed by the generation of the double mutant CBS7435-HyPer7∆*aox1*∆*aox2* (Mut-).

### Fluorescence microscopy

CBS7435-HyPer7 cells were inoculated in YPD medium (20 g/l peptone, 10 g/l yeast extract, 20 g/l glucose, pH 7.4–7.6) containing 200 µg/ml of hygromycin and incubated overnight at 25°C and 180 rpm.

On the following day, cells were inoculated in ASMv6 minimal synthetic media (Zavec et al. 2020) and 20 g/l of glucose. Inoculation started at an OD_600_ = 0.5 and cell were incubated until an OD_600_ of ~2–3 was reached. Cells were then either treated with 0.6 mM H_2_O_2_ or a control solution of phosphate buffered saline (1x PBS) for a duration of 20 min. Proceeding treatment, microscopic analysis was carried out using a Zeiss Axio Observer 7, equipped with filters for excitation at 405 and 488 nm. Images were analysed and processed using ImageJ.

### Microbioreactor cultivation

Microscale cultivations were performed in a BioLector I (M2p-Labs/Beckman Colter) employing three different filter modules. A 620 nm excitation filter was used in order to access scattered light and biomass levels. Predominantly oxidized HyPer7 signal was measured using a 488 nm excitation/520 nm emission while a 400 nm excitation/510 nm emission filter was used for measuring the reduced form.

All experiments were carried out in flower plates at 25°C and 1200 rpm. Measurements cycles were performed every 20 min. Final volume of media was of 1 ml per well.

### Effect of exogenous stressors

Initially, three individual clones of CBS7435-HyPer7 were precultured in selective YPD medium and incubated at 25°C, 180 rpm for 24 h. On the following day, using shake flasks, cells were inoculated in 10 ml of ASMv6 with 20 g/l glucose, at a starting OD_600_ = 0.5. Cells were then incubated in the same conditions until they reached an OD_600_ = 3 during exponential growth phase.

Once exponential phase was reached, cells were diluted to an OD_600_ = 0.5 in the same growth medium and inoculated into BioLector flower plates. Three hours into the experiment, H_2_O_2_ or dithiothreitol (DTT) were added in concentrations ranging from 0.1 to 20 mM. Additionally, lower concentrations of H_2_O_2_ (0.001–0.1 mM) were tested.

### Cultivation with different carbon sources

Cultivations comparing wild type and knockout strains were adapted from a previously described small scale screening process aiming for the selection of high protein-producing *K. phaffii* clones (Zavec et al. 2020). Briefly, individual clones were inoculated in 2 ml of selective YPD using 24 deep-well plates and incubated for 24 h at 25°C and 280 rpm. Subsequently, grown cells were inoculated in 1 ml of ASMv6 supplemented with 25 g/l of EnPump200 polysaccharide (Enpresso) and 0.35% glucose-release enzyme (Enpresso). This approach promotes a slow release of glucose, allowing for a small-scale simulation of fed-batch processes. Methanol was added to the culture with an initial 0.5% shot after 3 h, followed by the addition of 1% methanol shots at 19, 27, and 43 h.

In the case of methanol-only cultivations, a similar procedure was followed with slight modifications. Cells were inoculated into the BioLector flower plates with an OD_600_ = 0.5, and the initial medium containing only 1% methanol as carbon source. Subsequently, 1% methanol shots were administered after 40 and 65 h.

## Results

### Expression and response of HyPer7 to exogenous H_2_O_2_ in *K. phaffii*

**Figure 1.**
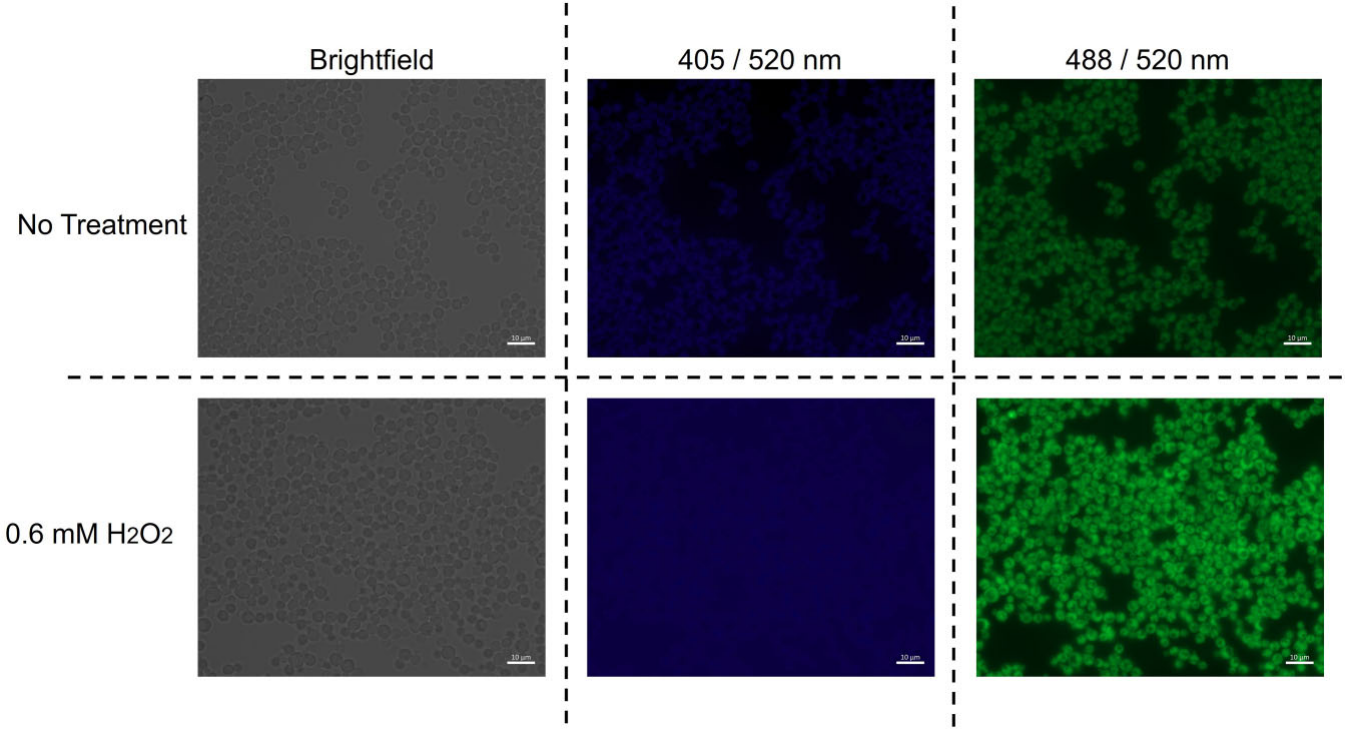
Microscopic comparison of CBS7435 *K. phaffii* expressing HyPer7 without any added stressor (upper row) and when treated with 0.6 mM of H_2_O_2_ for 20 min (lower row). Cells were analysed using brightfield (left column) and when excited at 405 nm (middle column) and 488 nm (right column), corresponding respectively to the reduced and oxidized forms of HyPer7. Quantitative fluorescence analysis of the images was performed for the AF488 filter using the ZEN software, and verified that the average intensity was higher for the cells treated with H_2_O_2_ (shown in Fig. S1).

HyPer7 was chosen to be expressed in the cytosol of *K. phaffii* strains, with the TEF promoter selected to ensure strong and constitutive expression. Subsequent phenotypic analysis of cells was performed using fluorescence microscopy, with excitabilities set at 488 nm and 405 nm for the recording, respectively, of the predominantly excited and reduced forms of HyPer7 (Fig. 1). The fluorescence intensity at 488 nm excitation exhibited a significant increase when cells were exposed to 0.6 mM H_2_O_2_ for 20 min suggesting that HyPer7 was not only correctly expressed, but also was responsive to H_2_O_2_ (Fig. 1 and Fig. S1).

### Cultivation of HyPer7 expressing *K. phaffii* in a BioLector allows in-line monitoring of H_2_O_2_ accumulation and decomposition dynamics

Once *K. phaffii* strains showing the correct expression and activity of HyPer7 were obtained, we aimed for analysing cellular responses upon exposure of cells to ROS or redox-active compounds. As redox-related events, such as the generation and accumulation of H_2_O_2_, are highly dynamic, tools that allow for a real-time monitoring are desired to avoid overlooking responses happening on a short time-scale. Having this in mind, cells were cultivated in a BioLector microbioreactor system, which allows for the monitoring of the fluorescence signals over the time-course of a cultivation. Once the signals for excitation at 488 and 400 nm were recorded, they were divided for the calculation of the Ox/Red (488/400) ratio.

### Response of HyPer7 to exogenous H_2_O_2_ and DTT

**Figure 2.**
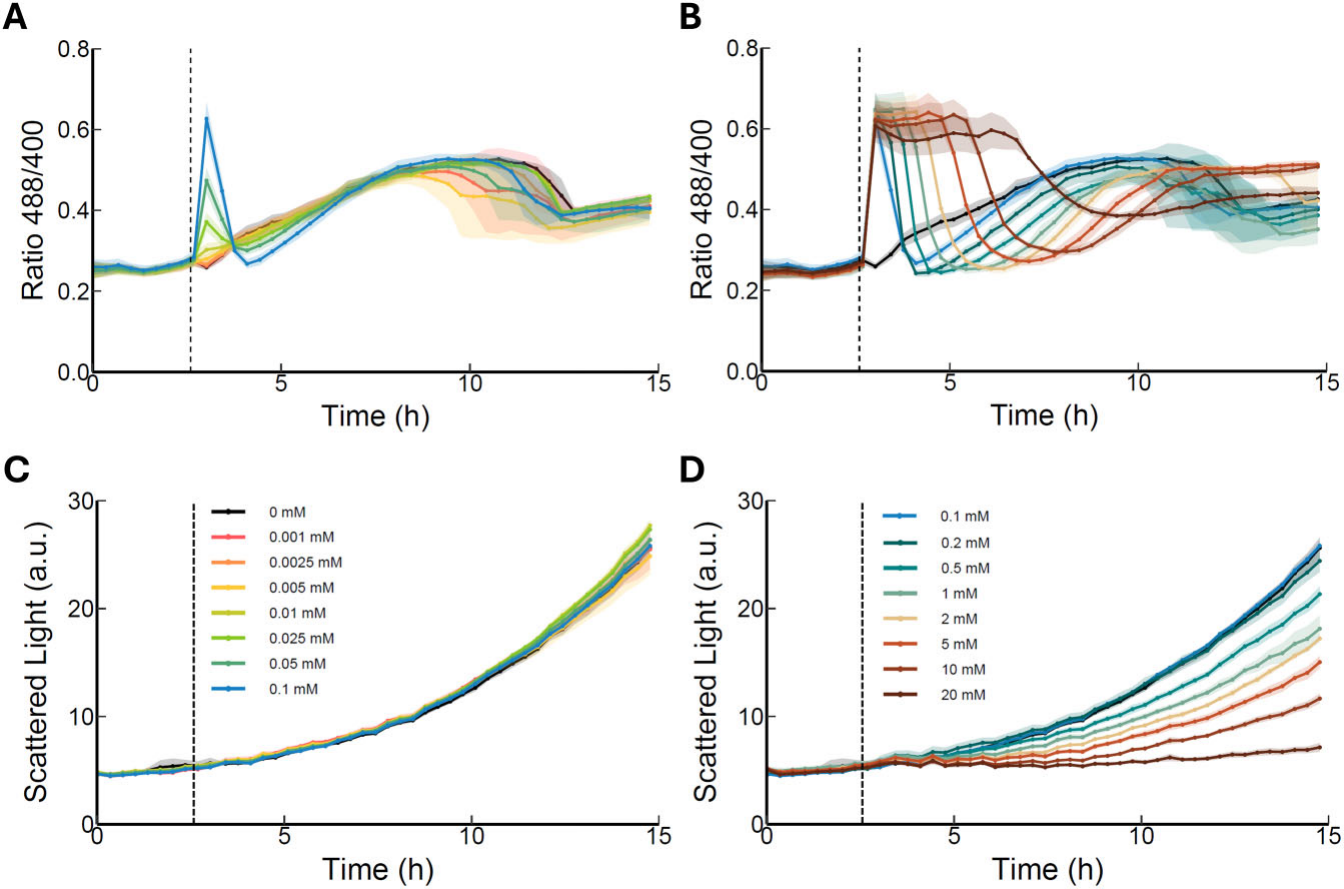
Response of yeast cells expressing HyPer7 to exogenously added redox stressors. Fluorescence signal ratio (A and B) and biomass signal (C and D) after addition of 0.001 to 0.1 mM H_2_O_2_ (A and C) or 0.1–20 mM H_2_O_2_ (B and D) to the media 3 h after cultivation started. Biomass is monitored by scattered light signal in arbitrary units (a.u.). Presented data is based on individual triplicates (*n* = 3). Solid lines are mean of triplicates. Shadowed region represents the standard deviation between replicates. Dashed vertical line represents the time point when the stressor was added. Individual signals of 488 (ox) and 400 (red) nm are given in Fig. S2.

Initially, cells were cultivated and analysed for their response to exogenously added stressors. As a reference control, *K. phaffii* HyPer7 cells grown without the addition of any stressor, exhibited no increase in the Ox/Red ratio during the first hours. During prolonged cultivation, the Ox/Red ratio started to increase, which is most likely due to respiratory activity during cell growth (black line, Fig. 2). To induce oxidation of HyPer7, H_2_O_2_ was added to the culture media 3 h after the start of cultivation, with concentrations ranging from 0.001 to 20 mM, and the fluorescence signals were followed. A noticeable change in the redox profile was observed, with a minimum concentration of 0.01 mM H_2_O_2_ already causing a significant increase in the 488/400 ratio when compared to the untreated cells (Fig. 2A). The ratio peaked after the addition of 0.25 mM H_2_O_2_ and exhibited a similar pattern across replicates: a sharp peak increase, followed by a decrease to basal levels within 3–5 h after addition of H_2_O_2_ (Fig. 2B). Whereas the individual Ox/Red ratio signal level including maximum peak height seemed to be instrument dependent factors, the relative patterns of response to H_2_O_2_ remained stable when measured at different Biolector instruments (Fig. S3). Similarly to the nontreated cells, the initial peak is followed by a subsequent approximately two-fold increase in signal ratio during prolonged cultivation (Fig. 2).

Interestingly, with increasing concentrations of H_2_O_2_, it took longer for the ratio to decrease from the first peak to the basal levels, and consequently, the second peak occurred later during the cultivation (Fig. 2). The maximum HyPer7 Ox/Red signal observed plateaued already upon the addition of 0.5 mM H_2_O_2_ and did not increase when higher levels of H_2_O_2_ were added, even following the addition of 20 mM H_2_O_2_. In contrast, when using the alternative biosensor roGFP2-Prx1, the maximal Ox/Red ratio increased continuously with the level of H_2_O_2_ added (Fig. S4), suggesting that HyPer7 expressed in *K. phaffii* is well tuned to measure low endogenous levels of H_2_O_2_ but gets saturated following the addition of high levels of external H_2_O_2_. Cells treated with higher concentrations of H_2_O_2_ experienced growth arrest (Fig. 2D), which was resumed only after 7–10 h, coinciding with the appearance of the second peak, again suggesting that this increase in H_2_O_2_ is related to cellular growth. Taken together, this clearly indicates that cultivating cells in the BioLector allows for in-line monitoring of the cellular response elicited by exogenous H_2_O_2_ addition. In this case, it is clear that concentrations of H_2_O_2_ between 0.01 and 0.2 mM were quickly degraded by the cellular antioxidant systems, as evidenced by the fast increase and decrease of the 488/400 fluorescence ratio, minimally affecting cellular biomass. On the other hand, at higher concentrations, results indicate that the cells take longer to decompose this stressor, therefore the ratio remains in a plateau, and cell growth was significantly affected.

Additionally, the response of HyPer7 to the addition of DTT was investigated (Fig. S3). While cells treated with 0.1 mM DTT grew similarly to the untreated cells, cell growth gradually decreased at higher DTT concentrations up to 1 mM, and was completely abolished above (5–20 mM DTT) (Fig. S3D). Importantly, the addition of DTT resulted in a small decrease in the ratio between the two analysed fluorescence signals already at 0.1 mM (Fig. S3B and E). This indicates that the disulfide bonds of the probe are being reduced by the action of DTT, resulting in an increase of the reduced forms of HyPer7, and suggest that HyPer7 can detect low levels of H_2_O_2_ produced by endogenous metabolism in unstressed cells.

### Accumulation of H_2_O_2_ changes drastically in cells grown with different carbon sources

To determine the effectiveness of the HyPer7 biosensor in evaluating the endogenous generation of H_2_O_2_ as a consequence of cellular metabolism, additional cultivations in the BioLector system were carried out. Given that *K. phaffii* is a methylotrophic yeast able to metabolize methanol to produce energy and biomass, monitoring cell redox state during this process is of high interest. The initial metabolic step that converts methanol into formaldehyde via the action of the Aox enzymes, results in the stoichiometric production of H_2_O_2_ according to enzymatic assays (Sahm and Wagner 1973, Couderc and Baratti 1980). However, so far the dynamics of this process in living cells remained unresolved.

**Figure 3.**
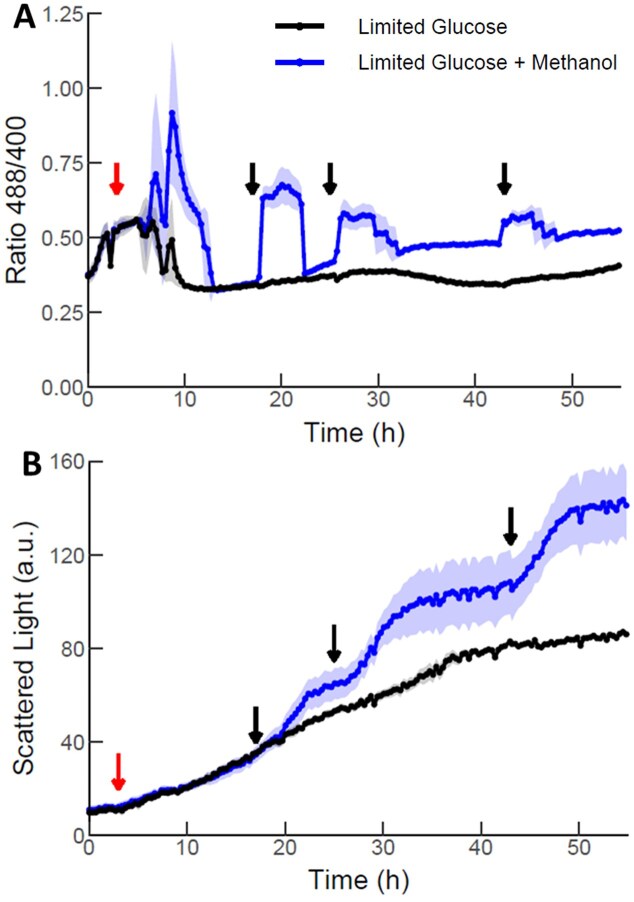
Redox and growth profiles of *K. phaffii* when cultivated in limiting glucose alone or with methanol addition as carbon sources. The addition of methanol leads to a higher accumulation of H_2_O_2_ (A) and an increase in the growth rate of cells (B). Biomass is monitored by scattered light signal in arbitrary units (a.u.). Presented data is based on individual triplicates (*n* = 3). Solid lines are mean of triplicates. Shadowed region represents the standard deviation between replicates. Red and black arrows represent, respectively, the addition of 0.5% and 1% methanol.

Therefore, our goal was to evaluate H_2_O_2_ accumulation recorded by the biosensor in conditions that mimic glucose-based or methanol-induced fed batch cultivations in the BioLector. Cells growing under glucose-limited conditions were compared to those subjected to methanol injections at various time points. Limited-glucose supply was ensured by providing a glucose polymer as carbon source, together with a glucose-releasing enzyme (EnPresso). Since *K. phaffii* is unable to metabolize the polysaccharide, glucose availability in the media is dictated by the activity of the releasing enzyme, simulating a fed-batch process. Again, an initial increase in the H_2_O_2_ signal was observed under both limiting-glucose conditions and cells treated with 0.5% methanol, which returned to a basal level ~10 h after start of cultivation or methanol addition, respectively (Fig. 3A). The cells induced with methanol reacted to each further methanol shot with an instant increase in the 488/400 fluorescence ratio, while the ratio remained at the basal level in glucose-limited cells until the end of the cultivation (Fig. 3A). After some time, the HyPer7 ratio decreased again, as the added methanol is consumed. The scattered light signal (Fig. 3B) increased exponentially after addition of methanol, representing cell growth. At the same time as the HyPer7 peaks decline, the scattered light signal remains constant (similar to stationary phase of a growth curve), indicating that cell growth ceases until the addition of the next methanol shot. This observation validates the production of H_2_O_2_ attributed to the activity of Aox enzymes upon MUT *in vivo*. Interestingly, the maximal height of the peaks became lower following subsequent methanol shots whereas, on the other hand, the signal did not return to basal levels later during cultivation. The lower maximal peak height might be explained as adding 1% methanol to a growing culture (i.e. increasing cell densities) results in decreasing amounts of methanol added per cell, and thus lower H_2_O_2_ generation.

### Knockout of Aox coding genes changes the redox profile of strains

The ability of *K. phaffii* to utilize methanol as sole carbon source is one of the main reasons of the popularity of this host organism. Recent sustainability considerations have rejuvenated the interest in organisms able to utilize C1 carbon sources (Kuzman et al. 2025). The first metabolic step of methanol metabolism in *K. phaffii* is carried out by two Aox isoforms, Aox1 and Aox2. This process uses O_2_ as the final electron acceptor, generating H_2_O_2_ as a by-product. In this study, three different genotypes were studied regarding their MUT: Mut^+^ strains containing both isoforms, Mut^S^ strains having the Aox1 isoform deleted (S stands for slow) and Mut^−^  *K. phaffii* strains lacking both isoforms, rendering them unable to grow using methanol as the sole carbon source (although they still consume methanol as energy source through the action of an alcohol dehydrogenase; Zavec et al. 2021).

**Figure 4.**
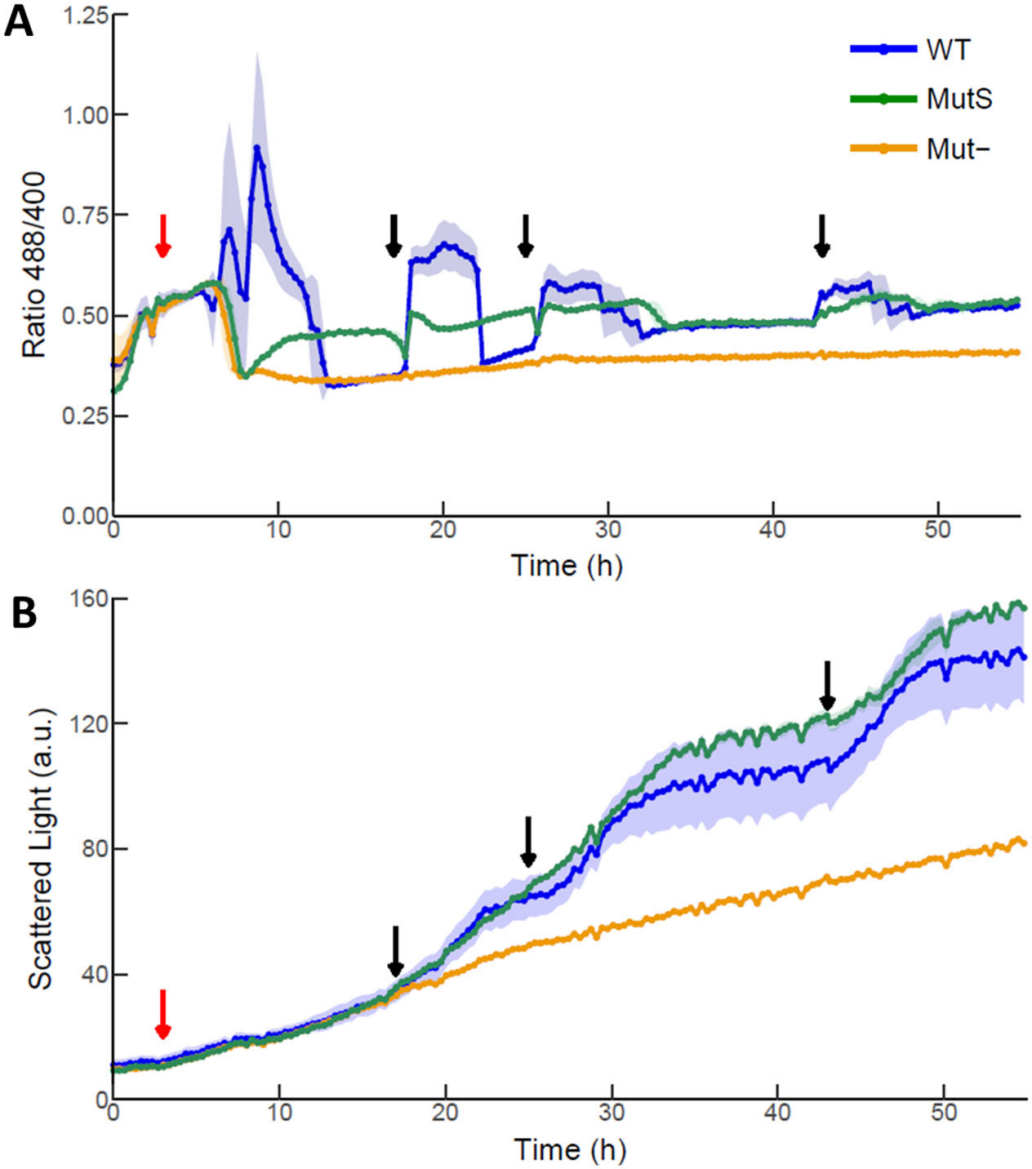
Effect of the lack of main methanol oxidizing enzymes (Aox1 and Aox2) on *K. phaffii* H_2_O_2_ accumulation (A) and growth (B). Cultivation of cells with a limiting glucose and methanol mix show that the accumulation of H_2_O_2_ is significantly higher in WT (Mut^+^) strains when compared to single *aox1∆* (Mut^S^) and double *aox1*∆ *aox2*∆ (Mut^−^) deletion strains. Biomass is monitored by scattered light signal in arbitrary units (a.u.). Presented data is based on individual triplicates (*n* = 3). Solid lines are mean of triplicates. Shadowed region represents the standard deviation between replicates. Red and black arrows represent, respectively, the addition of 0.5% and 1% methanol.

**Figure 5.**
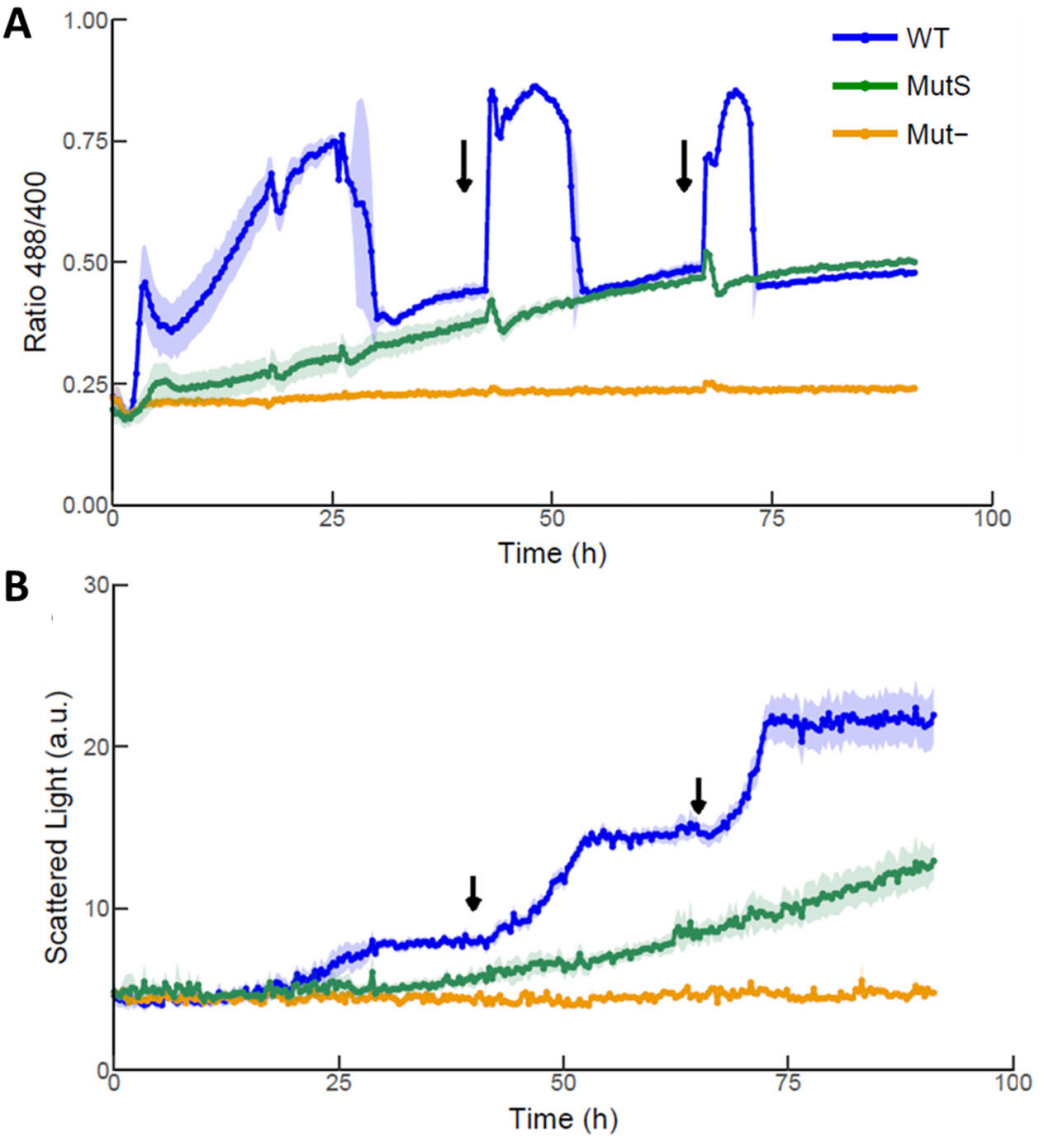
Effect of the lack of main methanol oxidizing enzymes (Aox1 and Aox2) on *K. phaffii* H_2_O_2_ accumulation (A) and growth (B) on methanol as sole carbon source. Cultivation of cells using methanol as sole carbon source show that the accumulation of H_2_O_2_ (A) is significantly higher in WT (Mut^+^) strains when compared to single *aox1∆* (Mut^S^) and double *aox1*∆ *aox2*∆ (Mut^−^) deletion strains. Biomass (B) was monitored by scattered light signal in arbitrary units (a.u.). Biomass was also significantly different between the strains, with the Mut^S^ and Mut^−^ presenting the adequate slow and lack of growth using methanol as carbon source, respectively. Presented data is based on individual triplicates (*n* = 3). Solid lines are mean of triplicates. Shadowed region represents the standard deviation between replicates. Arrows represent the addition of 1% methanol.

Mut^S^ and Mut^−^ strains were generated through CRISPR/Cas9-mediated knockout of respective genes in the CBS7435 Hyper7 reporter strain. Cells were then cultivated either under mixed carbon conditions using limited glucose with the addition of methanol shots at different timepoints (Fig. 4), or when only methanol was provided as sole carbon source for the whole experiment (Fig. 5). Under mixed carbon conditions, the growth of the Mut^−^ strain (yellow line, Fig. 4B) was comparable to the CBS7435 wild type strain grown on limiting glucose (black line, Fig. 3B). Indeed, also the redox state of HyPer7 in Mut^−^ strains in the mixed feed cultivation (yellow line, Fig. 4A) was highly comparable to the CBS7435 wild type strain grown in limited-glucose without methanol (black line, Fig. 3A). In contrast, no change in the redox signal was observed when the Mut^−^ strain was cultivated in media with methanol as sole carbon source, indicating that cells lacking both isoforms of Aox do not produce H_2_O_2_ (Fig. 5A). As expected, Mut^−^ strains did not show any growth when only methanol was available (Fig. 5B).

Comparisons between the Mut^+^ (wild type) and Mut^S^ strains clearly highlight the effects of *AOX1* knockout on cell growth and H_2_O_2_ production. Under mixed feed conditions, Mut^S^ cells lacking *AOX1* presented a decreased peak of the 488/400 ratio after each methanol addition indicating lower conversion of methanol into formaldehyde and H_2_O_2_ in addition to the subsequent reduction of the H_2_O_2_ produced (Fig. 4A). As both glucose and methanol are present, it is more difficult to directly correlate the generation of biomass (Fig. 4B), however, at later stages of cultivation, it is possible to observe that the addition of methanol confers higher growth rates to Mut^+^ strains than Mut^S^ immediately after methanol addition. In contrast, higher overall biomass concentrations were reached with Mut^S^ strains, suggesting that Mut^+^ cells exhibiting a fast methanol assimilation suffer from H_2_O_2_ and formaldehyde toxicity which results in reduced biomass yield. Thus, the sensor allows bioprocess optimization to balance the rate of methanol assimilation against the toxicity of methanol oxidation products formed in the process.

This pattern was more pronounced when using methanol as the sole carbon source (Fig. 5A). With an initial concentration of 1% MeOH, Mut^+^ strains presented a continuous 488/405 peak from the start of cultivation, decreasing to lower levels after ∼30 h, coinciding with the cessation of growth (Fig. 5B). Upon addition of additional 1% MeOH shots, cells resumed growth, accompanied by an increase in the H_2_O_2_ detected. In contrast, Mut^S^ strains presented a significant lower growth rate when only methanol was present in the media, never reaching a point when cells ceased growth due to having used up all available MeOH (Fig. 5B). This growth pattern also correlated with the accumulation of significantly lower basal levels of H_2_O_2_, as well as peak heights in response to methanol pulses (Fig. 5A).

**Figure 6.**
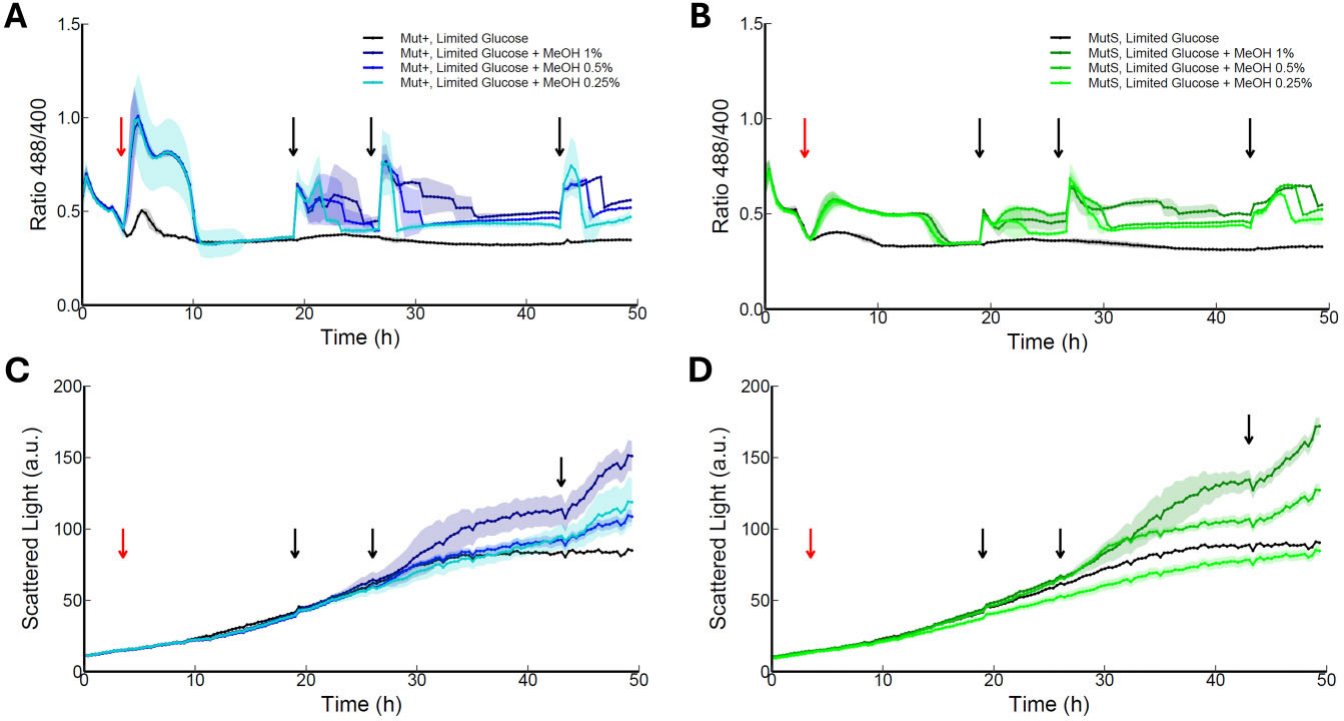
Effect of adding different concentrations of methanol (MeOH) on H_2_O_2_ accumulation and growth of *K. phaffii* Mut^+^ and Mut^S^. At 3 h of cultivation on limiting glucose (see Fig. 3), cells were induced with 0.5% methanol (red arrows). Black arrows indicate the time of addition of different concentrations of MeOH (0.25%, 0.5% or 1%; black arrows) to the Mut^+^ wildtype (A and C) and the Mut^S^ strain (B and D). The HyPer7 signal ratio (A and B) returned faster when lower methanol concentrations were added. Biomass (C and D) was monitored by scattered light signal in arbitrary units (a.u.). Presented data is based on individual triplicates (*n* = 3). Solid lines are mean of triplicates. Shadowed region represents the standard deviation between replicates.

To further investigate the correlation between MUT and the generation of H_2_O_2_, the experiments were repeated with the Mut^S^ and Mut^+^ strains growing in limiting glucose and following the addition of different levels of methanol (Fig. 6). After the initial 0.5% methanol shot (red arrow), three different concentrations of methanol were added in the three later timepoints. Both strains showed a similar pattern as described before (Fig. 4) when adding repeated shots of 1% methanol. When adding lower concentrations of methanol (three times 0.5%), the HyPer7 signal ratio reached the same height as for 1% methanol, but declined to basal levels faster (Fig. 5A and B). This effect was even more pronounced when 0.25% of methanol was added. As expected, biomass was lower when lower amounts of methanol were added (Fig. 6C and D). This again confirms that the generation of H_2_O_2_ is arising from the activity of the Aox enzymes during MUT.

## Discussion

The control of the redox state by cells is crucial for a healthy functioning of living organisms, with a variety of different players and reactions involved. The generation and accumulation of ROS is essential for proper functioning of cell metabolism and signaling, while at higher levels also causing cellular damage (Knoefler et al. 2012). Monitoring ROS, specially H_2_O_2_, is becoming increasingly popular with the use of genetically encoded biosensors, such as HyPer and roGFP-based tools (Belousov et al. 2006). Both sensors are dynamically reflecting probe oxidation by H_2_O_2_ and probe reduction by intracellular antioxidant defense systems. Such indicators have been used in different organisms, such as plants (Dopp et al. 2023), mammalian cells (Pak et al. 2020), fission yeast (De Cubas et al. 2021), and baker’s yeast (Kritsiligkou et al. 2021, Gast et al. 2022).

Here, we applied HyPer7 for the first time in *K. phaffii*, a methylotrophic yeast which is popular for recombinant protein production in biotechnology and industry. Similarly to previous work in *S. cerevisiae* (Kritsiligkou et al. 2021), HyPer7 was shown to exhibit bright fluorescence when expressed using a strong promoter (TEF), demonstrating full functionality also in the nonconventional yeast *K. phaffii*. The combination of HyPer7 expressing strains with the cultivation in a microbioreactor (BioLector, mM2p-labs/Beckman Coulter Life Sciences) allowed real time monitoring of the biosensor redox state during yeast growth (Gast et al. 2022) and provided an appropriate setup for following the dynamics of H_2_O_2_ generation and accumulation in various contexts.

The probe also responded to the addition of DTT (Fig. S3). At low DTT concentrations, a less oxidized signal ratio was observed, suggesting that HyPer7 is also suited for the detection of low levels of H_2_O_2_ produced endogenously during unstressed growth of *K. phaffii* in normal growth medium. Remarkably, HyPer7 showed an increased oxidation signal upon addition of intermediate DTT concentrations (0.5–1 mM), indicating that cytosolic H_2_O_2_ levels temporarily increase upon reductive stress (Fig. S3). Reductive stress induced by the addition of intermediate levels of DTT (5 mM) has, perhaps a bit surprisingly, previously been shown to elicit a Yap1/Skn7-dependent antioxidant response and to increase both oxygenation and ROS levels in *S. cerevisiae* (Maity et al. 2016). This response was proposed to shut down protein synthesis in response to the accumulation of oxidatively unfolded proteins in the ER, in line with the proposed role of H_2_O_2_ in inhibiting translation (Topf et al. 2018). Our data suggests that this response is most probably also conserved in *K. phaffii*.

Furthermore, HyPer7 responded to low concentrations of exogenously added H_2_O_2_ (with a limit of detection of 0.01 mM H_2_O_2_ in the analysed conditions), showing a peak in the 488/405 ratio, that was not observed in untreated cells. The peak was quickly reduced, highly comparable to what was previously observed in *S. cerevisiae* (Kritsiligkou et al. 2021). In addition to HyPer7, we also tested the roGFP2-based sensors roGFP2-Prx1 [containing *S. cerevisiae* Prx1 (Van Laer and Dick 2016, Gast et al. 2022)] and roGFP2-Tsa2dCr (Morgan et al. 2016) expressed under the same strong P_TEF_ promoter, which have shown higher sensitivity in *S. cerevisiae* and *S. pombe* (De Cubas et al. 2021, Kritsiligkou et al. 2021). Unexpectedly, roGFP2-Tsa2dCr exhibited photobleaching in the microscope and both showed significantly lower sensitivity for H_2_O_2_ than HyPer7 in *K. phaffii* (Fig. S4), and where thus excluded for further studies. However, even though roGFP2-Prx1 only responded to higher H_2_O_2_ concentrations (>1 mM) in our experiments, it returned faster to its reduced state than HyPer7, and might thus be suited for specific applications where higher H_2_O_2_ levels need to be monitored in *K. phaffii*.

Increased concentrations of exogenously added H_2_O_2_ showed that complete oxidation of the HyPer7 sensor was achieved after the addition of 0.25 mM. Untreated cells exhibited a slight increase in oxidation during prolonged cultivation. This increase is likely correlated to H_2_O_2_ generated by cell growth (respiration). At higher concentrations (≥1 mM), H_2_O_2_ was responsible for arresting cellular growth, and as H_2_O_2_ levels decreased, cells regained their ability to grow, culminating in the appearance of a second peak, mirroring untreated cells (Fig. S5). This reinforces the hypothesis that initially, H_2_O_2_ is generated as a byproduct of cellular growth, likely as a result of the main sources of ROS in the cell, such as mitochondria and the ER.

The methylotrophy of *K. phaffii* makes it one of the popular microbial hosts used for the production of recombinant proteins (Zahrl et al. 2017) and new C1-based technologies (Baumschabl et al. 2024). The methylotrophic phenotype is based on the presence of Aox enzymes, that initiate the conversion of methanol into formaldehyde by the oxidation of water into H_2_O_2_ (Yurimoto et al. 2011). Here, we monitored the dynamics and differences in the accumulation of H_2_O_2_ over time during cultivation of *K. phaffii* in a BioLector (m2p-labs/Beckman Coulter Life Sciences) using a previously described screening method for small scale assessment of *K. phaffii* production strains (Staudacher et al. 2022). Our experiments revealed a sharp increase in H_2_O_2_ levels immediately following the addition of methanol to the culture. By analysing our data it is also possible to identify that especially Mut^+^ cells go through a period of starvation between methanol shots, indicating that the methanol feeding regimen could be even further optimized by decreasing the intervals between the methanol shots to avoid starvation. Previously, accumulation of ROS in *K. phaffii* has been determined at distinct timepoints offline using different redox sensitive dyes (Delic et al. 2014, Cai et al. 2022). Even though the use of such dyes may be very informative, there remain uncertainties mainly due to their limited specificity for distinct ROS molecules and their overall low sensitivities (Murphy et al. 2022). Here, we show that the changes in the H_2_O_2_ levels can be very abrupt and rapid, making it extremely advantageous to use a genetically encoded biosensor, since it avoids the need of manipulating the cells and specific sampling limitations inherent to redox staining. Furthermore, in-line measurements such as in the BioLector proved to be valuable to monitor the changes over time.

One of the main challenges of methylotrophic yeasts are the high oxygen requirements and the oxidative stress generated during methanol metabolism (Zavec et al. 2020). Among different methylotrophic yeasts, a different range of Aox isoforms are present (Ito et al. 2007). In *K. phaffii*, the MUT phenotype is conferred by two enzymes, Aox1 and Aox2 (Cregg et al. 1989). The two enzymes are almost identical in sequence, nevertheless Aox1 is believed to be the main isoform. To elucidate their contribution to H_2_O_2_ formation, we created Mut^S^ (*∆aox1*) and Mut^−^ (*∆aox1∆aox2*) strains by sequential deletion of both Aox coding genes using CRISPR/Cas9. The deletion of the *AOX1* gene resulted in substantially decreased levels of H_2_O_2_, whereas in the double mutant strain that cannot metabolize methanol the production of H_2_O_2_ was completely abolished. This shows that Aox1 indeed is the dominant methanol oxidizing enzyme and a major source of cellular H_2_O_2_.

## Conclusions

In conclusion, HyPer7 in combination with in-line monitoring by a BioLector was successfully set up for *K. phaffii* and proved to be a powerful tool for assessing longstanding topics, such as the first *in vivo* confirmation of the generation of H_2_O_2_ via methanol metabolism. Furthermore, it offers insights into overcoming bottlenecks in strain and bioprocess engineering. For example, we could clearly see why Mut^S^ strains outperform their Mut^+^ counterparts in screening conditions, as they allow for a more continuous activation of the *AOX* promoter and lower levels of the toxic by-product H_2_O_2_. This also opens a lot of opportunities for the future by studying cell physiology and production related stresses in *K. phaffii* using such biosensors e.g. the measurement of H_2_O_2_ levels as a consequence of the production of secretory recombinant proteins. Future research might involve also targeting of the biosensor to specific organelles to provide also spatially resolved information.

## Supplementary Material

foaf070_Supplemental_File
