# Supplementary material for: The genetically encoded biosensor HyPer7 enables in-line monitoring of H2O2 accumulation dynamics in the methylotrophic yeast Komagataella phaffii
Source: FEMS Yeast Res. 2025 Nov 22;25:foaf070. doi: 10.1093/femsyr/foaf070 (PMC12684170; doi:10.1093/femsyr/foaf070)
Supplement: foaf070_Supplemental_File [file foaf070_supplemental_file.pdf]

Supplementary File to

The genetically encoded biosensor HyPer7 enables in-line monitoring of low levels of endogenous H_2O_2 in the methylotrophic yeast *Komagataella phaffii*

Victor Mendes Honorato, Jennifer Staudacher, Mikael Molin, Brigitte Gasser

Supplementary Table S1: List of strains used in this work

Strain	Genotype	Resistance	Reference
**CBS7435 + HyPer7**	P_{TEF}-HyPer7	Hyg	This work
**CBS7435 Mut^S + HyPer7**	*Δaox1* P_{TEF}-HyPer7	Hyg	This work
**CBS7435 Mut$^-$+ HyPer7**	*Δaox1Δaox2* P_{TEF}-HyPer7	Hyg	This work
**CBS7435 + roGFP2-Prx1**	P_{TEF}-roGFP2-Prx1	Hyg	This work
**CBS7435 + roGFP2-Tsa2dCR**	P_{TEF}-roGFP2-Tsa2dCR	Hyg	This work

Supplementary Table S2: List of primers used in this work

Primer Name	Sequence 5´-> 3
**HyPer7 Fw**	GATCGGTCTCACATGCGTGGTTCTCACC
**HyPer7 Rev**	GATCGGTCTCAAAGCTCAATCACAGATGAATGAGAC
**roGFP2 Fragment 1 Fw**	GATCGGTCTCACATGGCTAGCGAATTCTCA
**roGFP2 Fragment 1 Rev**	GATCGGTCTCACCTTGAAATCGATACCCTTC
**roGFP2 Fragment 2 (M1) Fw**	GATCGGTCTCAAAGGAAGATGGTAACATCTTG
**roGFP2 Fragment 2 Rev**	GATCGGTCTCATTCATTTGGATCTTTGGATAAAG
**roGFP2 Fragment 3 (M2) Fw**	GATCGGTCTCATGAAAAGAGAGATCATATGGTATTG
**roGFP2 Fragment 3 (Linker) Rev**	GATCGGTCTCAGAATTCTCCTCCTGATCCT
**Linker Prx1 Fragment 1 Fw**	GATCGGTCTCAATTCTTTAGTAGAATTTGTAGCGCTC
**Prx1 Fragment 1 Rev**	GATCGGTCTCATGACCCATCATTGATATTTTTGAA
**Prx1 Fragment 2 (M1) Fw**	GATCGGTCTCAGTCACTGAAAACCGTGAG
**Prx1 Fragment 2 Rev**	GATCGGTCTCAAAGCTTATTTCGACTTGGTGAATCTT
**Linker Tsa2 Fw**	GATCGGTCTCAATTCGTAGCAGAAGTTCAAAAACAAG
**Tsa2 Rev**	GATCGGTCTCAAAGCTTAATTATTGGCATTTTTGAAATACTC

Supplementary Table S3: List of plasmids generated in this work

Plasmid	Gene/<br>OE Cassete	Resistance	Integration locus
**BB1-HyPer7**	HyPer7	Kan	-
**BB1-roGFP2-PRX1**	roGFP2-PRX1	Kan	-
**BB1-roGFP2-TSAdCR**	roGFP2-TSAdCR	Kan	-
**BB3-HyPer7**	P_{TEF2}-HyPer7	Hyg	*ENO1*
**BB3-roGFP2-Prx1**	P_{TEF2}-roGFP2-Prx1	Hyg	*ENO1*
**BB3-roGFP2-Tsa2ΔCR**	P_{TEF2}-roGFP2-Tsa2ΔCR	Hyg	*ENO1*

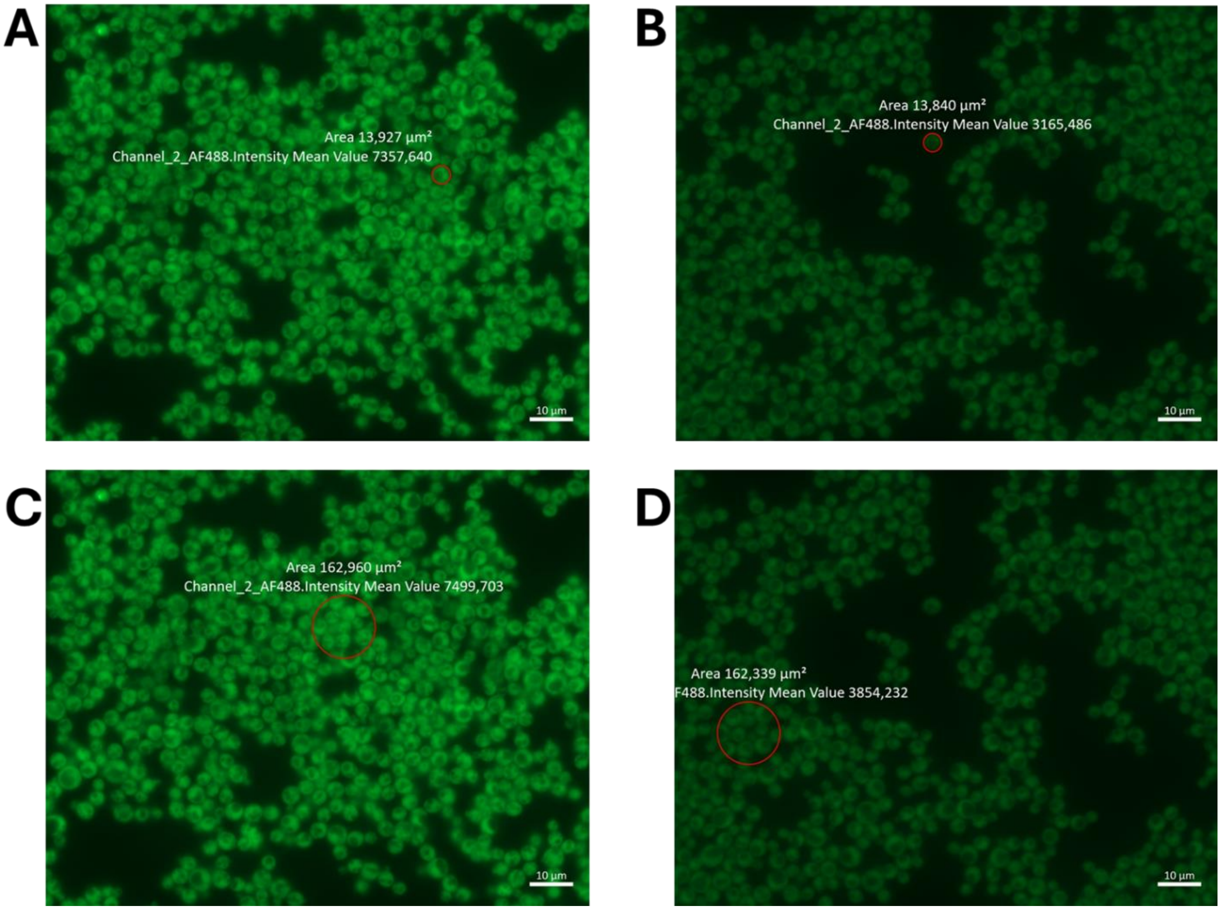

Figure S1: Quantitative image analysis of *K. phaffii* expressing HyPer7 with and without added H_2O_2. Microscopic fluorescence quantification of a single cell (A, B) or a group of cells (C, D) of the images shown in **Figure 1** was performed for the AF488 filter using the ZEN software. The average fluorescence intensity was higher for cells treated with 0.6 mM H_2O_2 for 20 min (A, C) than for cells without any added stressor (B, D).

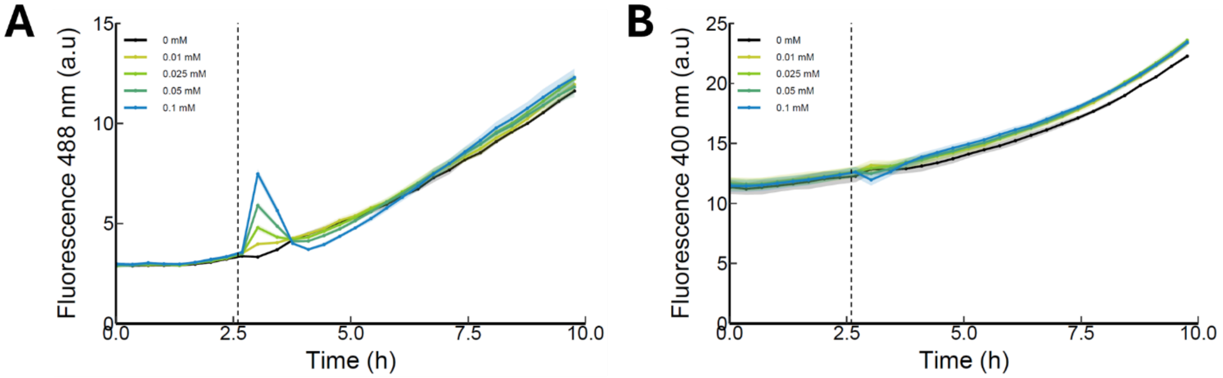

Figure S2: Response of yeast cells expressing HyPer7 to exogenously added H_2O_2. Fluorescence signals at 488 nm and 400 nm after addition of 0.01 to 0.1 mM H_2O_2 to the media 3 h after cultivation started. The presented data is the raw data to Figure 2 and based on individual triplicates (n = 3). Solid lines are mean of triplicates. Shadowed region represents the standard deviation between replicates. Dashed vertical line represents the time point when the stressors were added.

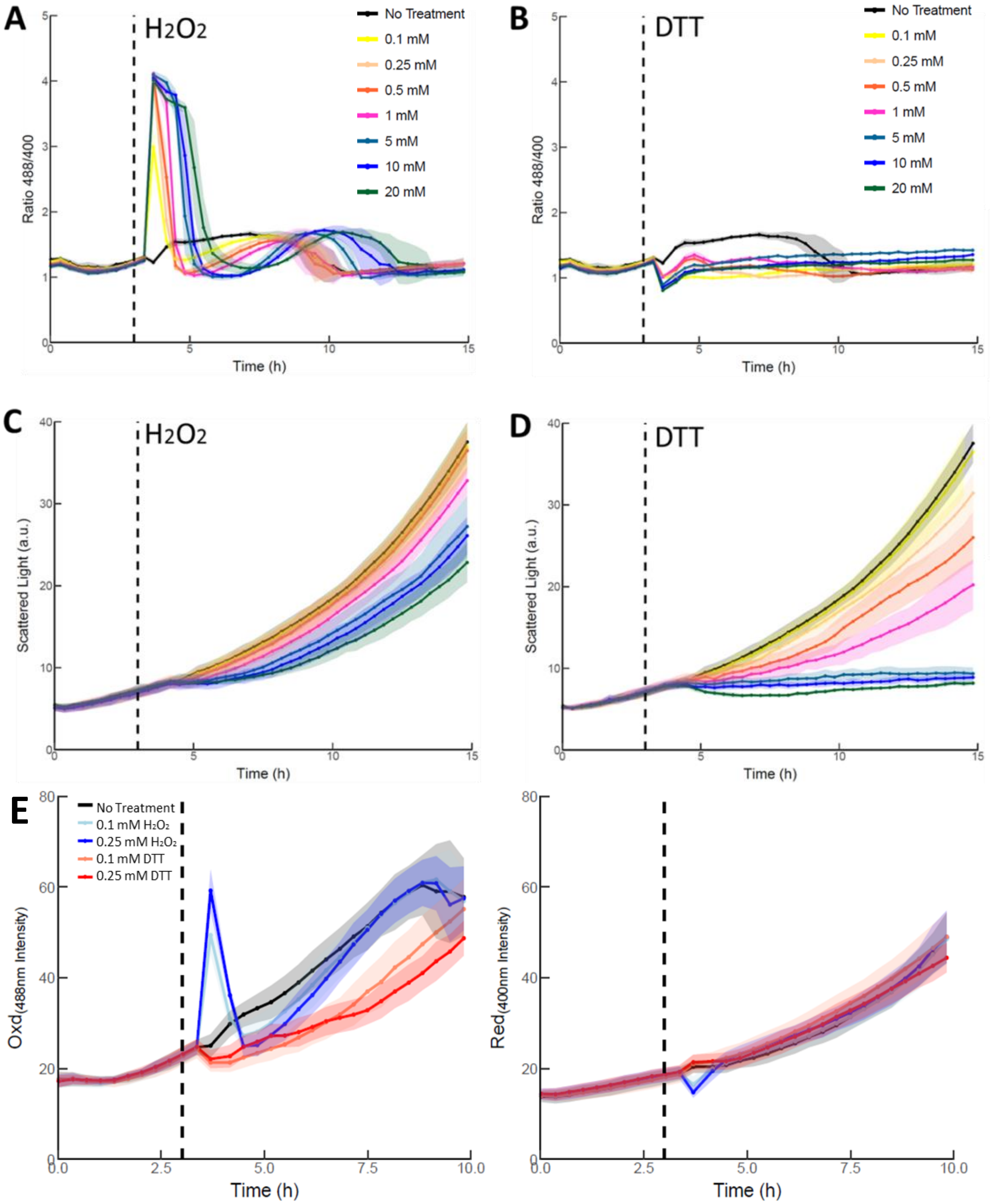

Figure S3: Response of yeast cells expressing HyPer7 to exogenously added redox stressors. Fluorescence signal ratio (**A**, **B**) and biomass signal (**C**, **D**) after addition of 0.1 to 20 mM H_2O_2 (**A**, **C**) or 0.1 to 20 mM DTT (**B**, **D**) to the media 3 h after cultivation started. Biomass is monitored by scattered light signal in arbitrary units

(a.u.). Presented data is based on individual triplicates (n = 3). Solid lines are mean of triplicates. Shadowed region represents the standard deviation between replicates. Dashed vertical line represents the time point when the stressors were added. **E**) Individual intensity of the signals at 488 (ox) and 400 (red) nm for 0.1 and 0.25 mM added stressor.

A

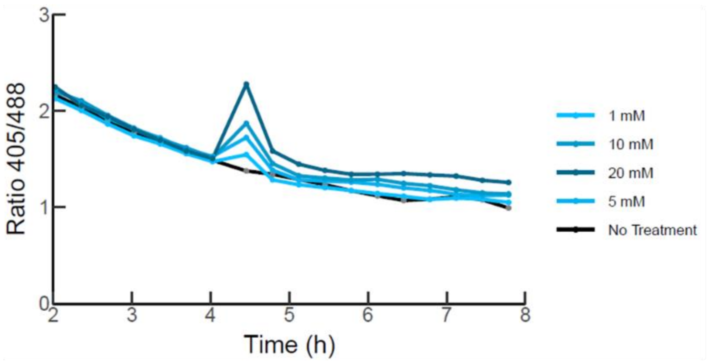

B

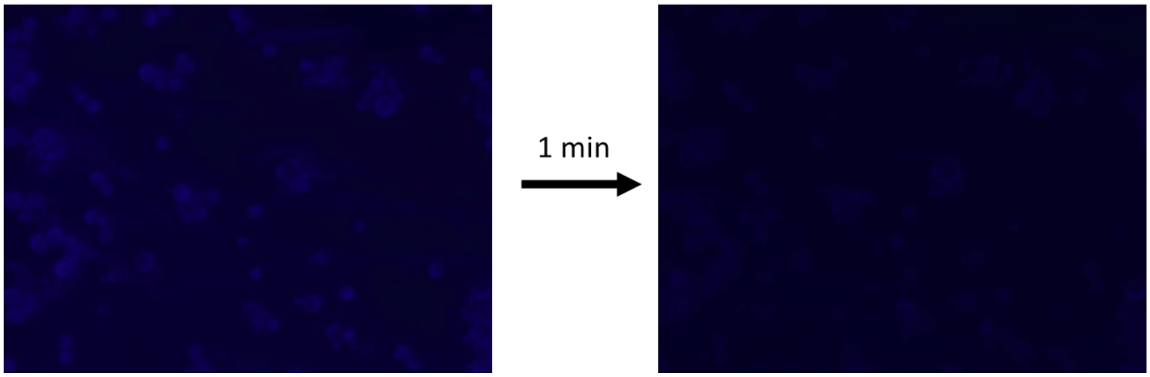

Figure S4: Application of roGFP2 based biosensors in *K. phaffii*. A) Response of CBS7435 roGFP2-Prx1 to increasing concentrations of H_2O_2. B) Microscopic analysis of CBS7435 roGFP2-Tsa2dCr revealed fluorescence bleaching after 1 minute of observation.

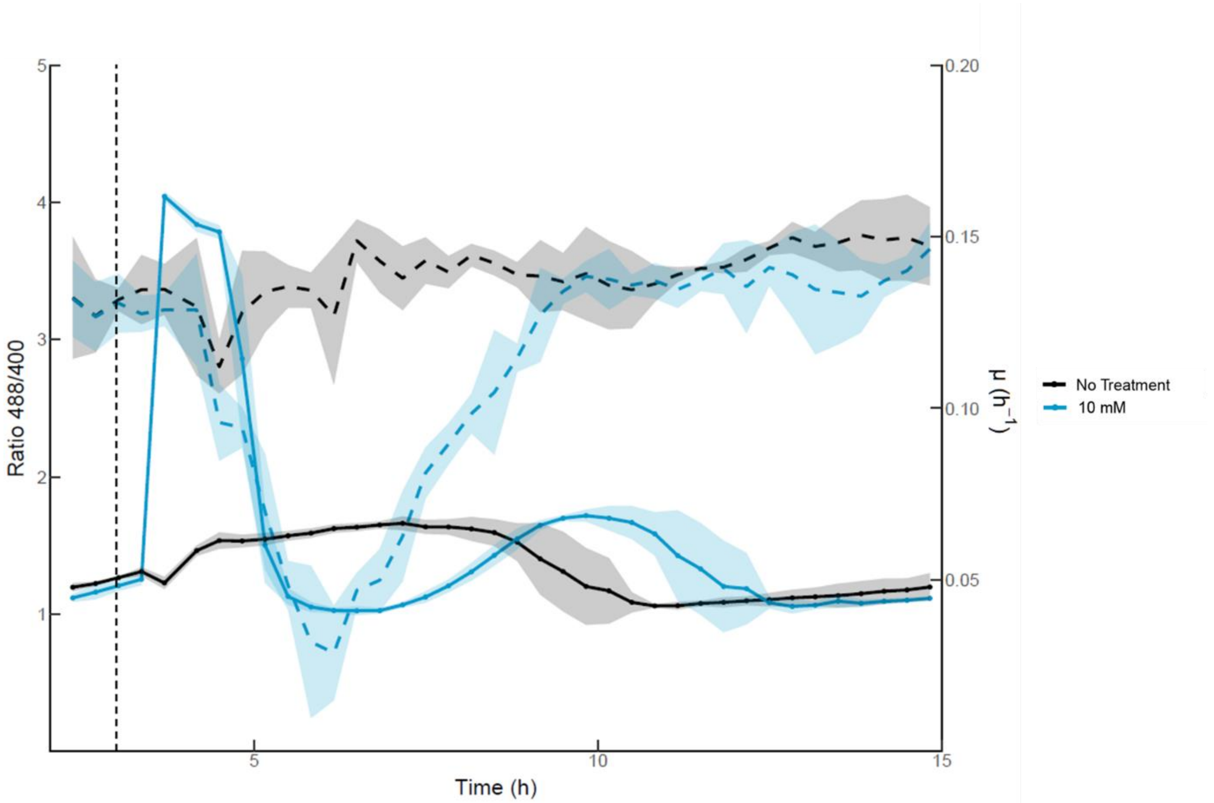

Figure S5: Correlation between the growth rate of CBS7435-HyPer7 (dashed line) and the redox state of the biosensor (solid line). Dashed vertical line represent the addition of 10 mM of H_2O_2.
